# Graphene for the Building of Electroanalytical Enzyme-Based Biosensors. Application to the Inhibitory Detection of Emerging Pollutants

**DOI:** 10.3390/nano11082094

**Published:** 2021-08-18

**Authors:** Marta Bonet-San-Emeterio, Noelia Felipe Montiel, Manel del Valle

**Affiliations:** Sensors and Biosensors Group, Department of Chemistry, Universitat Autònoma de Barcelona, Edifici CN, 08193 Bellaterra, Spain; marta.bonet@uab.cat (M.B.-S.-E.); Noelia.FelipeMontiel@uantwerpen.be (N.F.M.)

**Keywords:** graphene, biosensing, inhibition, phenols, laccase enzyme

## Abstract

Graphene and its derivates offer a wide range of possibilities in the electroanalysis field, mainly owing to their biocompatibility, low-cost, and easy tuning. This work reports the development of an enzymatic biosensor using reduced graphene oxide (RGO) as a key nanomaterial for the detection of contaminants of emerging concern (CECs). RGO was obtained from the electrochemical reduction of graphene oxide (GO), an intermediate previously synthesized in the laboratory by a wet chemistry top-down approach. The extensive characterization of this material was carried out to evaluate its proper inclusion in the biosensor arrangement. The results demonstrated the presence of GO or RGO and their correct integration on the sensor surface. The detection of CECs was carried out by modifying the graphene platform with a laccase enzyme, turning the sensor into a more selective and sensitive device. Laccase was linked covalently to RGO using the remaining carboxylic groups of the reduction step and the carbodiimide reaction. After the calibration and characterization of the biosensor versus catechol, a standard laccase substrate, EDTA and benzoic acid were detected satisfactorily as inhibiting agents of the enzyme catalysis obtaining inhibition constants for EDTA and benzoic acid of 25 and 17 mmol·L^−1^, respectively, and a maximum inhibition percentage of the 25% for the EDTA and 60% for the benzoic acid.

## 1. Introduction

A chemical sensor is defined by IUPAC as a device that transforms chemical information into an useful analytical signal [1]. The use of sensors has progressed over the years with the constant development of new technologies and devices. Researchers try to improve the selectivity of these designs with a wide range of techniques, such as the modification of the transducer material, the use of chemometric data processing, or the inclusion of sample pretreatment. An example of the former is the concept of biosensors. A biosensor may be described as a sensor combined with a recognition element from the biological world, which is the one responsible for conferring paramount selectivity. Actually, this is what makes the difference from common sensors and grants their success. Since the discovery of graphene and its derived products, they have been gaining importance in the sensors and biosensors field [2]; one of the reasons for this is due to their synergistic effect when they are implemented as transducing materials [3,4]. It can be proven that the usage of graphene is becoming widespread when you search how many publications exist with “graphene” as a keyword, since 2004, when graphene was strictly discovered. Almost 150,000 publications can be listed in total; from these, 14,117 can be found using the “graphene + sensor” keyword combination (data according to Scopus database).

Among others, graphene and its derivates have gained popularity in electroanalysis due to their interesting inherent properties, such as biocompatibility, low-cost raw materials, and their easy tuning [3,4]. In this work, graphene is used not only as a key material to enhance the response of a bare electrochemical sensor, but also to show its properties as a linkage platform for enzyme immobilization [5,6,7]. Graphene allows to avoid sensor pretreatment or modifications that can be costly, time-consuming, and complex. One example is electrochemical grafting, where the carboxylic groups of an organic layer are attached to a conductive surface [8,9,10].

Graphene synthesis can be classified into two groups: top-down, and bottom-up synthesis. Furthermore, new strategies may be mentioned in the state of the art of the topic; for example, the cracking of multiwalled carbon nanotubes to create graphene oxide (GO)ribbons [11,12]. Bottom-up syntheses are the ones that start from simple carbon structures to create a pristine single-atom graphene layer with minimum defects and a *sp2* configuration. Some examples of bottom-up approaches are the chemical vapor deposition (CVD) or the epitaxial growth [13,14]. Nevertheless, these synthetic pathways have important drawbacks, such as the complex adaptation to large scale production and the expensive and complex arrangements [15]. For this reason, top-down syntheses are popular when large amounts of materials are required. Hummers’ synthesis is the most frequently used method for the top-down production of graphene [16,17]. This wet synthesis uses strong oxidizing agents for exfoliating graphite to GO. As it is known, GO is a hydrophilic nonconductive material, which needs further synthetic steps to recover the pristine graphene properties, one of them the conductivity, an essential feature in electrochemistry [15,17]. Thus, a further step is needed, the GO conversion to the reduced graphene oxide (RGO). This step can be done electrochemically, chemically, mechanically, or even thermally. Even though, in the sensors study area, the most used reduction techniques are the electrochemical and the chemical [18,19,20]. Both methods are interesting due to their reproducibility and easy applicability; however, the electrochemical approach presents several clear advantages over the chemical approach, e.g., low-cost, speed, environmentally friendliness and simplicity [20]. Therefore, in this work, RGO has been produced electrochemically. This method is based on the reduction of oxidized moieties present on the GO surface. As an additional advantage, the electrochemical reduction allows to easily and effectively control the oxidation degree of the RGO by simply adjusting the applied potential, which may be interesting for different final applications [21,22].

Graphene is not the unique strategic nanomaterial used to enhance the sensors response. For example, carbon dots and nanotubes have been also widely used as electrode modifiers. Carbon dots, also named graphene quantum dots or carbon nanodots, are a new trend in electroanalysis applications. Up to recent times, this material has been used mainly in photoluminescence-based determinations; but in current years it is growing strongly also in the electrochemical field [23,24,25,26]. On the other side, carbon nanotubes, considered as a rolled layer of graphene [27], have been extensively studied as a sensor modifier as well. Like other materials from the carbon family, carbon nanotubes ascribe catalytic effects and sensing improvements to the electrode behavior [28,29,30].

Enzymes such as laccase, tyrosinase, and glucose oxidase have been employed for a long time in bioanalytical chemistry, especially in biosensing. Their relevance is related to their ability to interact only with their matching substrates, conferring to the device one of the most desired characteristics: the specificity and the ability to distinguish the analyte in complex matrices. One of the most famous case is the glucose biosensor, already commercialized as a device for diabetic patients [31,32]. On the other side, laccase, a protein with a copper nucleus, has been extensively studied as a biorecognition element in various areas owing to its ability to oxidize a wide range of phenolic compounds, being catechol one of the standards for its activity analysis [33,34]. But not only there have been studies on the direct interaction with the substrates, but also on the interaction of the enzymes with their inhibitors. An interesting example is its use in detecting pollutants in products for human consumption, especially in drinking water. UNESCO reports as a pollutant any synthetic or naturally occurring chemical or microorganism that is not commonly monitored or regulated in the environment. This group includes pharmaceuticals, personal care products, pesticides, industrial additives, and industrial and household products [35]. New pollutants, also named emerging pollutants or contaminants of emerging concern (CECs), are appearing continuously. These chemicals have become an increasing problem over the years. Fortunately, at the same time, technology has been developed which has introduced new and improved methods to isolate and/or detect CECs in human consumption products. However, the existing commercial techniques are time-consuming and expensive, which encourages researchers to find new alternatives. Moreover, it must be considered that at this moment there are no reference methods in the European legislation to control the presence of these compounds in water. Nevertheless, different factors will force their consideration in the near future. The first reason is the conclusions from recently published articles, which determine that certain CECs, in special endocrine disruptors, interfere in the human and animal endocrine system [36,37,38]. The second reason is that these pollutants are not only found in water but also in soil and marine species such as shellfish and oily fish, increasing the contact of humans with these compounds with still unknown long-term effects. This work reports the preparation and characterization of a laccase-graphene biosensor for the detection of CECs (EDTA and benzoic acid) by the inhibition of the laccase enzyme.

## 2. Materials and Methods

### 2.1. Chemical and Reagents

All reagents were of an analytical grade and all solutions were prepared using deionized water from Milli-Q system (Millipore, Billerica, MA, USA). Samples for electrochemical measurements were prepared on phosphate buffer in the presence of KCl as a background electrolyte, reagents purchased from Merck KGaA (Darmstadt, Germany).

For the graphene synthesis, it was used as a graphite powder (50 µm, sulfuric acid (98%), sodium nitrate (99%), and potassium permanganate (99%), purchased from Merck KGaA (Darmstadt, Germany); hydrogen peroxide (30%) was purchased from Riedel-de Haën (Germany). Used ionic resins were strong cationic resin C100E and weak anionic resin A520E (Purolite) purchased from Merck KGaA. Ba(NO_3_)_2_ was purchased at Acros Organics (Geel, Belgium).

The material needed for the building of the electrode were: Resineco Epoxy Kit 125 resin supplied from Resineco green composites (Barcelona, Spain) and graphite powder (particle size < 50 μm) was received from BDH (BDH Laboratory Supplies, Poole, UK). For the biosensor, Laccase from *Agaricus Bisporus*, 7.2 U·mg^−1^ (EC number: 420-150-4), 1-ethyl-3-(3-dimethylaminopropyl carbodiimide (EDAC), and N-hydroxysulfosuccinimide (sulfo-NHS) (purchased from Merck KGaA) were used.

Finally, catechol, acid benzoic, and EDTA were purchased from Merck KGaA.

### 2.2. Electrochemical Measurements

Voltammetric measurements were performed on a AUTOLAB PGSTAT30 (Ecochemie, Netherlands) controlled by GPES software. Electrochemical Impedance Spectroscopy (EIS) experiments were performed on a Bas-Zahner IM6e (Kronach, Germany) potentiostat controlled by Thales software. For both electrochemical techniques a typical three electrode circuit was used. In the used assembly it can be found: the working electrode based on a graphite epoxy composite (electrode already optimized in our laboratory [39,40,41]), an Ag/AgCl (0.1 mol·L^−1^ KCl) electrode as a reference and a Pt electrode as a counter. All measurements were carried out at room temperature without stirring using as an electrolyte solution phosphate buffer 50 mmol·L^−1^ + KCl 100 mmol·L^−1^ at pH 7.4.

Parameters for the cyclic voltammetry (CV) measurement were optimized for its use; at the end, a scan rate of 10 mV·s^−1^ and a step potential of 0.001 V was applied. The analysis of CV was performed with the GPES software, in particular using the inset tool to calculate the base line and the corresponding peak height of the recorded data. 

### 2.3. Sensor Characterization

Morphological characterization of the raw materials was carried essentially by Transmission Electron Microscopy (TEM); TEM images were obtained using a JEOL JEM-2011 microscope. Moreover, X-ray photoelectron spectroscopy (XPS) analysis was carried out to describe the structure of the synthetized GO; a SPECS PHOIBOS 150 hemispherical energy analyzer with monochromatic Cu–Kα radiation was used. CasaXPS software was used to acquire the fitted values.

Surface roughness assessment was done by processing of topographic atomic force microscopy (AFM) images obtained using a Veeco Dimension 3100 AFM Microscope (Bruker, Billerica, MA, USA).

### 2.4. Graphene Synthesis and Characterization

A top-down synthesis based on a modified Hummers’ method was used for the synthesis of GO [16,18,42]. In short, the protocol consists of the addition of 1.5 g of graphite and 4.5 g of NaNO_3_ to 35 mL of sulfuric acid (98%) cooled at 0 °C. Afterwards, 4.5 g of KMnO_4_ in fine powder form was added in small portions over a period of 2 h, avoiding temperature from rising and the mixture was stirred for an additional 4 h. The resulting mixture was heated at 35 °C for 30 min and the oxidation process was completed by slowly adding 75 mL of deionized water. In this highly exothermic step, it is important to avoid boiling. The reaction finishes 15 min after being kept at 70 °C. In order to eliminate unreacted oxidizing compounds, the solution was treated with 300 mL of 10% hydrogen peroxide. The resulting brown paste (the densest GO) was washed multiple times with deionized water, recovering the solid by centrifugation at 6000 rpm. This process was repeated several times, till the pH approached neutrality. With a pH close to 7, the sample of GO was treated with cation and anion exchange resins to eliminate the remaining salt impurities. This was done just by placing in contact, under vigorous stirring, the sample and resin for 30 min. The separation was achieved by using a 50 μm sieve. Sulfate ions were eliminated with Ba(NO_3_)_2_. Finally, the obtained slurry was dried in a vacuum oven at 60 °C for 48 h.

### 2.5. Graphene Oxide Modification and Immobilization of the Enzyme for the Biosensor Building

**Figure 1:**
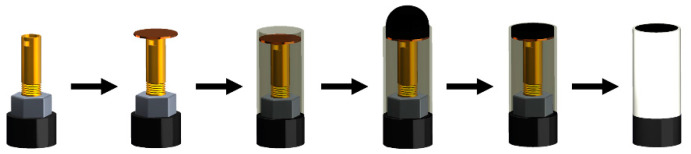
Scheme of the mechanic assembly for the construction of the base graphite-epoxy voltammetric transducer.

The graphite epoxy composite (GEC) electrode, used as base transducer, had an average geometric area of 28 mm^2^ (Ø = 6 mm), as it is specified in the reference bibliography [39,40,43]; the composite conductive paste was added in an assembly formed by an electrical connector and a PVC tube. Afterwards, the conductive material was hardened in an oven at 40 °C. Finally, the electrode was polished with different grain sandpapers until a homogeneous and pristine surface was obtained. The polishing step can be applied when needed to regenerate the surface and obtain again the bare platform. Figure 1 sketches the construction process.

Once the conductive element of the proposed biosensor was ready, it was modified with GO. The selected technique to modify the electrode surface was drop casting, based on the physical adsorption of the GO to the base carbon material. Firstly, 40 µL of the GO dispersion (1 mg·mL^−1^ in deionized water) was deposited on the GEC electrode. It may be noted that the GO suspension must be sonicated previously for 1 h and centrifugated for 1 min at 800 rpm to use only the part of the dispersion that content the flakes with fewer layers. After this, the electrode was dried at 40 °C to promote physisorption. The RGO was obtained by the electrochemical reduction of the previous electrode. As it was first optimized [43], the reduction was carried out via CV; in particular, 10 cycles were applied in a potential range going from +1.90 V to −2.30 V at 0.1 V·s^−1^.

The laccase enzyme was covalently bonded to the RGO via carbodiimide (EDAC, N-(3-dimethylaminopropyl)-N′-ethylcarbodiimide hydrochloride) chemistry. The EDAC reaction proved to be the most suitable strategy to immobilize the enzyme since it is able to react (through a free amino group) with the remaining carboxylic groups from RGO (that were not reduced in the reduction step) to obtain the amide bond. Concretely, after the reduction, a solution of 10 mg·mL^−1^ of laccase in water was prepared, where 100 µL of EDAC solution (1000 ppm) and 50 µL of sulfo-NHS solution (1000 ppm) were added. Once the solution was homogenized, the RGO electrode was incubated overnight in an Eppendorf tube [44,45,46,47]. The last step was to clean the non-bonded compounds from the surface of the electrode by rinsing it for 1 h in the working phosphate buffer.

**Figure 2:**
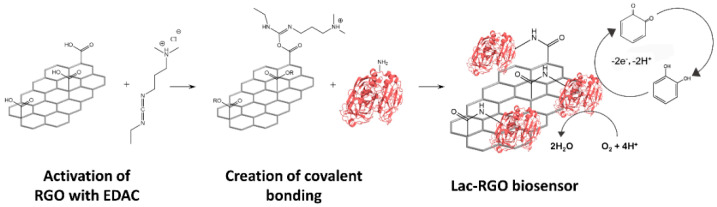
Scheme of the laccase immobilization. Step 1: activation of the RGO using EDAC (and NHS to increase the efficiency), Step 2: bonding creation between the primary amine from the enzyme and the intermediate complex of the RGO surface. Finally, the catalytic reaction is described.

As Figure 2 depicts, the EDAC coupling agent is used to activate the carboxylic groups from the RGO surface and form an intermediate complex that can react with the primary amine group from the laccase chain. In this article, sulfo-NHS was additionally used to obtain a more stable and efficient intermediate complex.

## 3. Results

### 3.1. Study of the Synthesized Graphene Oxide

**Figure 3:**
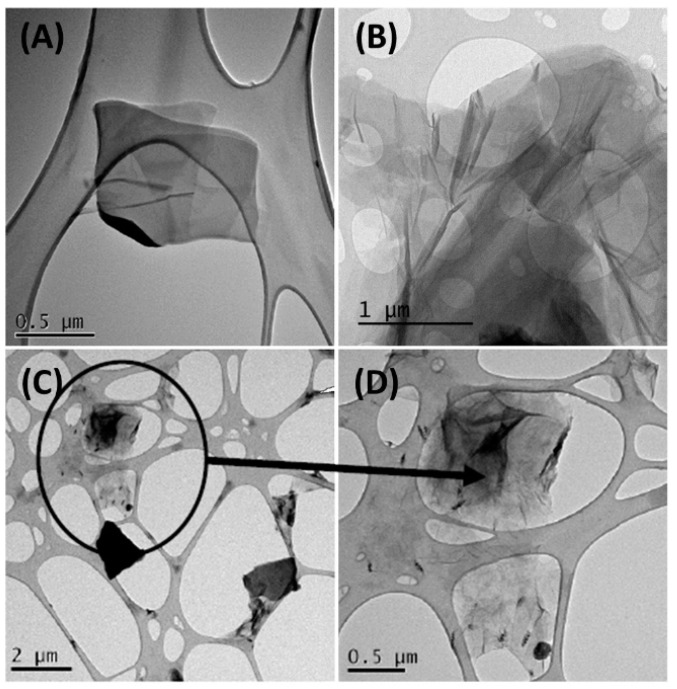
TEM images of synthesized GO. The samples were suspended in ethanol absolute (≥99%) and casted on a CuTEM grid. (**A**,**C**) show entire flakes of graphene oxide meanwhile in figures (**B**,**D**) it is presented a specific part where graphene characteristics can be spotted.

The obtained GO was characterized by TEM. Figure 3 shows some representative images resulting from this technique. Herein, the main part of graphene flakes was thin and transparent enough to see the CuTEM grid through them. The presence of GO was corroborated when its characteristic wrinkles were identified, present, for example, in Figure 3A,B,D [48,49]. The main presence of few layers of aggregates, which is supported because not only the transparency is achieved but also clear borders, is also noticed. Particularly, in Figure 3A,D can be seen clear and sharp edges, whereas in Figure 3B the presence of a large number of layers can be deduced from the multiple grey shades at the edge of the flake. These hypotheses were compared with the literature, where other researchers have similar TEM images [22,50,51]. The opaque materials in Figure 3C may be associated with the graphite residues coming from the starting synthesis reagents.

**Figure 4:**
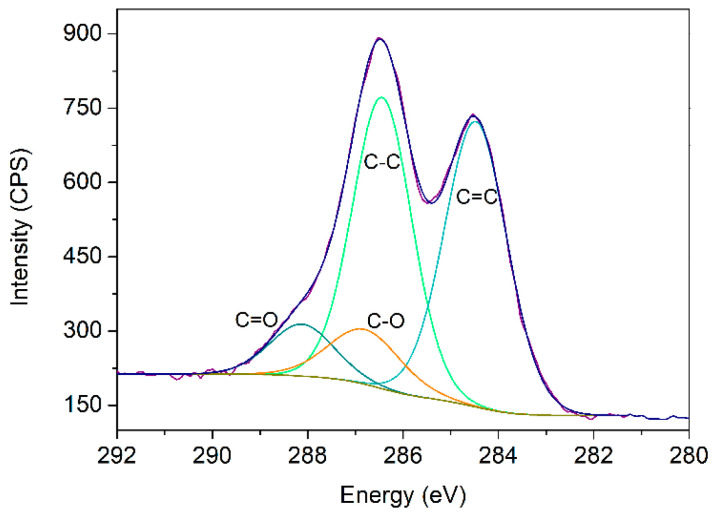
GO-Fitted XPS spectra of C 1s and its deconvolution. Blue line corresponds to the fitted curve.

In addition to TEM, XPS analysis was carried out to study the obtained GO (Figure 4). Table 1 presents the dominating groups assigned to C 1s core level after its deconvolution. Different groups could be found in the material, corresponding to the wide range of oxygen functional groups from the synthetic pathway. To confirm this hypothesis, the oxygen and carbon ratio was calculated, being equal to 2.14. Normally, the approved ratio values range between 2.1 and 2.9 for GPO samples [16].

Briefly, the resulting material of the modified Hummers’ method has similar properties and characteristics to comparable works in the literature [17,20,22,52], reflecting then the good performance.

### 3.2. Modification and Characterization of the Graphene Sensor

**Figure 5:**
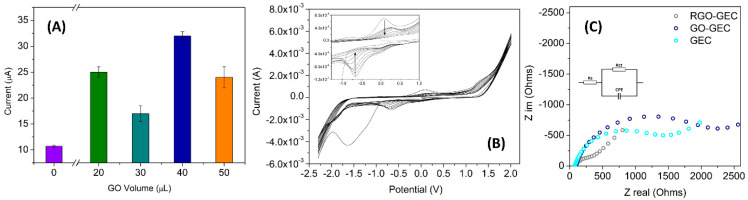
Optimization of the critical parameters for the use of the sensor. In (**A**) the deposited volume of GO via drop casting (*n* = 3) (**B**,**C**) activation step of GO to RGO. (**B**) depiction of the 10 cycles reduction step; (**C**) shows the Nyquist plot after the reduction of GO.

The optimization of the sensor assembly was carefully addressed. First, the optimal amount of GO solution to be deposited onto the electrode was studied. In this case, drop-casting was chosen as the modification method, due to its rapid and easy application. The effect of four different volumes (20, 30, 40, and 50 µL) of a 1 mg·mL^−1^ GO dispersion (in deionized water) was studied. As mentioned above, GO is not conductive. For this reason, after the deposition, it was reduced to RGO to analyze its response using electrochemical techniques. The analytical performance was studied by using a CV technique, by measuring the height of the oxidative peak at a potential of 0.18 V (vs. Ag/AgCl reference electrode) in the electrochemical standard [Fe(CN)_6_]^3-/4-^ 5 mmol·L^−1^ (diluted in phosphate buffer 50 mmol·L^−1^ at pH 7.4) as analyte. The goal was to find a volume that covers all the electrode surface and did not saturate the response. As can be observed in Figure 5A, the obtained results, together with visual checking, demonstrate that smaller volumes did not cover the whole area of the electrode, but at the same time volumes higher than 40 µL formed aggregates too large to be stacked in the surface for long periods of time, deteriorating any graphene benefits in the electrochemical responses. At the sight of these results, 40 µL was the selected volume for preparing the graphene-modified electrode.

**Figure 6:**
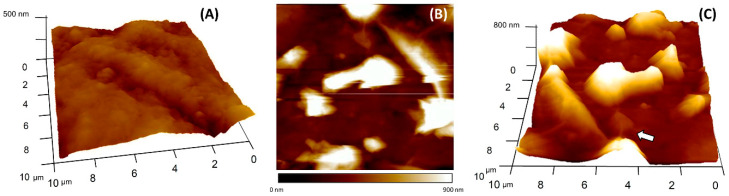
AFM images comparing (**A**) bare GEC electrode and (**B**) a GEC electrode modified with GO. (**C**) 3D AFM representation of the GO-GEC electrode.

The electrode surface was characterized also topographically via AFM, in order to visualize how the graphene was distributed onto the electrochemical platform. Figure 6A shows the surface of a bare GEC electrode, and Figure 6B,C is a representative region of a GO modified GEC electrode, all images have a scanned surface of 10 × 10 µm. As it can be seen in Figure 6A, the GEC electrode presents a crumpled surface with a root mean square roughness (R_RMS_) of 73.5 nm; meanwhile, the surface modified with GO has a R_RMS_ of 123.3 nm. Those values, along with the AFM images, verify the presence of graphene flakes. Moreover, the 3D AFM image (Figure 6C) indicates the presence of multiple and separated GO flakes, some of them clearly with less height than the others. Even in image 6C (marked with an arrow), it is possible to notice the GO wrinkles, also observed in TEM images (Figure 3). To sum up, AFM images demonstrated the correct stacking of GO, which lays in the surface increasing the rugosity, and consequently, the active sites of the electrode surface. 

One essential step for the building of the biosensor is the correct reduction of GO to RGO; in this work, the electrochemical reduction step was followed via CV, and verified with EIS by the comparison of the different surfaces. In Figure 5B, the CV plot corresponding to the electrochemical reduction of the GO to RGO is shown. Specifically, the good performance of the process was assessed through the oxidation and reduction peaks that appear in the range of −1.0 V to −0.5 V (vs. Ag/AgCl) (see insert on Figure 5B). According to the literature, these peaks are related to the reduction of oxygen groups present in the deposited GO in aqueous medium and neutral pH [20,53]. An EIS technique was also used to confirm the efficiency of the electrochemical reduction. The differences in the electrode surface of the sensors can give information on the electron transfer and thus demonstrates the increment in conductivity of the RGO. In this case, the impedance characterization experiments were carried out in [Fe(CN)_6_]^3-/4-^ 5 mmol·L^−1^ between 0.5 MHz and 0.1 Hz at a sinusoidal voltage perturbation of 10 mV amplitude. Figure 5C shows the Nyquist plot for each modification phase; the bare GEC electrode, the electrode when the GO material was deposited and finally when the last material, RGO, was obtained. In the same figure is inserted the Randles’ equivalent circuit, which includes: (i) Rs, the ohmic resistance of the bulk solution; (ii) Rct, the charge transfer resistance, which describes the difficulty of the electrochemical reaction; and (iii) CPE, a constant phase element, the component that gives information on the double layer capacitance. Figure 5C shows a smaller Rct for RGO-GEC electrode (206 Ω) than for GEC (1638 Ω) and GO-GEC (2256 Ω), indicating that the conductivity of graphene is regenerated with the electrochemical method applied in this work. Additionally, the data demonstrate the better conductivity of the RGO-modified electrode than the non-modified one.

These observations allowed us to confirm that GO is deposited correctly onto the GEC surface using 40 µL; moreover, it could be assumed that the reduction method is good enough to recover the conductive properties of graphene and proceed to covalently immobilize the enzyme via the EDAC reaction.

### 3.3. Characterization and Application of the Biosensor

#### 3.3.1. Electrochemical Response of the Laccase Biosensor

Once the enzyme was immobilized, EIS was used to compare and contrast the biosensor against the already-evaluated platform.

The results of Table 2 compare Rs, Rct and CPE values for the most significant steps of the electrode modification. The most characteristic value in impedance results is the Rct; in this case, it is easy to see the little difference between the electrodes, RGO (206 Ω) and the laccase-RGO (231 Ω) electrodes, which expose less resistance to the current flow than the bare one (1638 Ω). The effects seen in Rct of the laccase-RGO electrode could be explained with the negative charge formed in the carboxylic groups of the amino acids, which are deprotonated at pH 7.4, generating a minimal increase in the electronic transference between the electrode surface and the negative complex used as analyte. Moreover, CPE, a complex value with empirical significance, was adjusted. The results indicate changes that may be related to the double layer capacitance thickens the decrease. Rs, as mentioned above, describes the bulk features of the solution and the diffusion characteristics; therefore, it may not be affected by the modifications on the electrode surface as can be observed in the table, indicating the ideal situation.

**Figure 7:**
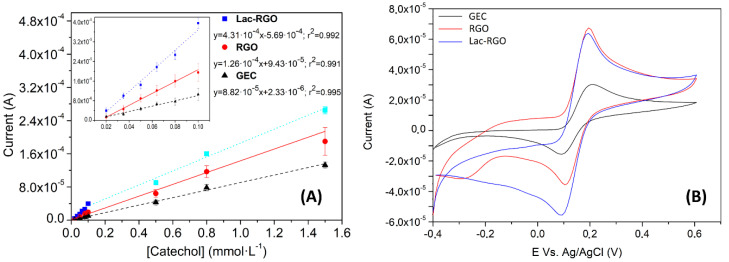
(**A**) Calibration curves for Lac-RGO electrode (∎), RGO electrode (●) and GEC electrode (▲) for the same concentrations of catechol. Currents for the oxidative peak (**B**) CV voltammograms response of the three electrodes in the same concentration of catechol. Measures in phosphate buffer + KCl solution, pH 7.4.

Finally, the resulting biosensor was tested versus a phenolic compound, catechol, which is a standard substrate of the laccase enzyme. In Figure 7A is shown the excellent performance of the enzyme by the comparison of the responses of each sensor against different concentrations of catechol. Note that the anodic peak was used as the analytical signal in the following experiments since, contrary to what was expected, it is the part where the synergistic effect of all the system elements was best [54,55,56]. In the calibration curves, observed a linear trend from 2 to 100 µmol·L^−1^ for the biosensor and a wider range for the bare and the RGO electrodes was observed, which goes from 2 to 1500 µmol·L^−1^ and from 2 to 800 µmol·L^−1^ respectively. In the same image is also noted a clear enhancement in the sensitivity of the system, increasing by more than threefold the initial slope value. 

Figure 7B compares GEC, RGO, and Lac-RGO responses against the same concentration of catechol. In the figure, as expected from the previous characterization, a current enhancement can be observed when the GEC electrode is modified with graphene. On the other side, some differences are appreciated when RGO and Lac-RGO responses are compared, which leads us to assume the laccase activity. Two of the most characteristic changes are observed in the oxidation baseline and the reduction peak. These facts are caused by the catalysis of the anodic reaction, and consequently there is a significant enlargement of the cathodic pathway.

#### 3.3.2. Analytical Characterization of the Biosensor

Once the biosensor operation was confirmed, their analytical properties were studied in detail. The properties studied were the linear range, limit of detection (LOD), limit of quantification (LOQ), repeatability, intermediate precision, selectivity, and lifetime.

The linear range is generally defined as the area in the calibration curve where the signals are directly proportional to the concentration. This parameter is typically evaluated using the correlation coefficient. As can be observed in the inset of Figure 7A, the linear range found spanned from 2 to 100 µmol·L^−1^. The correlation coefficient for this range was 0.992, indicating a satisfactory linearity.

Regarding the LOD and LOQ, there are multiple ways to calculate them, such as the use of the calibration curve for the target analyte (see Equation (1), where m is the slope of the calibration curve and σ the standard deviation of the response at a 95% confidence level), the measure of signal/noise ratio or by the use of the blank standard deviation [57,58]. In this work, we used the first option, obtaining an LOD of 2.1 µmol·L^−1^ and a LOQ of 7.7 µmol·L^−1^.

(1) $$\mathrm{LOD}\;=\frac{3.3\;\sigma }{m}\;\mathrm{LOQ}\;=\frac{10.0\;\sigma }{m}$$

**Figure 8:**
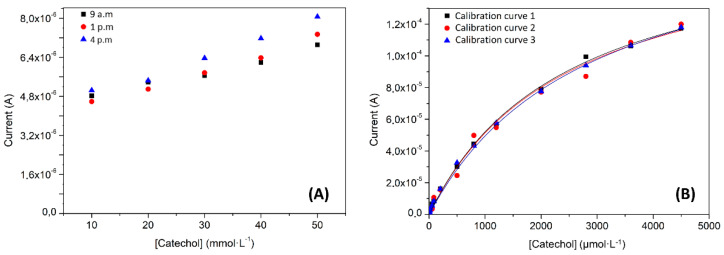
Study of the Lac-RGO analytical biosensor properties, (**A**) calibration curves repeatability test; measurements carried out in three-time instants and without regeneration of the electrode, (**B**) intermediate precision test made with 3 calibration curves on 3 different days with renewed electrode surface.

The repeatability is defined for the European Medicines Agency (EMEA) as the “term that expresses the precision under the same operating conditions over a short interval of time” [59]. In this case, 10 consecutive measurements of a 50 µmol·L^−1^ catechol solution were performed, obtaining a relative standard deviation (RSD) value equal to 8%. Moreover, a second repeatability test was done to check the reliability of the biosensor during the day. For this purpose, 3 calibration experiments were repeated in 3 different time instants of the day (9 a.m., 1 p.m. and 4 p.m., see Figure 8A). The results showed an RSD for each point of the curve not larger than 8%. All repeatability tests were taken as positive, considering that a group of data is significantly equal when the RSD value is lower than 10%. In the EMEA guideline, is also defined the intermediate precision [59]. This term expresses “the within-laboratories variations, for example, when considering different days of analysis, different analyst or analysis in different equipment”. This test measured, across three different days, three different calibration curves, from three regenerated biosensors, checking at the same time two parameters, the difference between days and the difference of the electrode surface regeneration procedure (see Figure 8B). The findings indicated that there were no differences between the regenerated electrodes or the measurements on different days (RSD = 6%). The last studied parameter was the lifetime of the biosensor (or the intermediate precision of different days). To perform this test, an electrode was stored in the fridge at 4 °C in a closed Eppendorf and a measurement was done every day (without regeneration of the surface) using a 50 µmol·L^−1^ catechol solution. The RSD results were 3% (*n* = 3).

**Figure 9:**
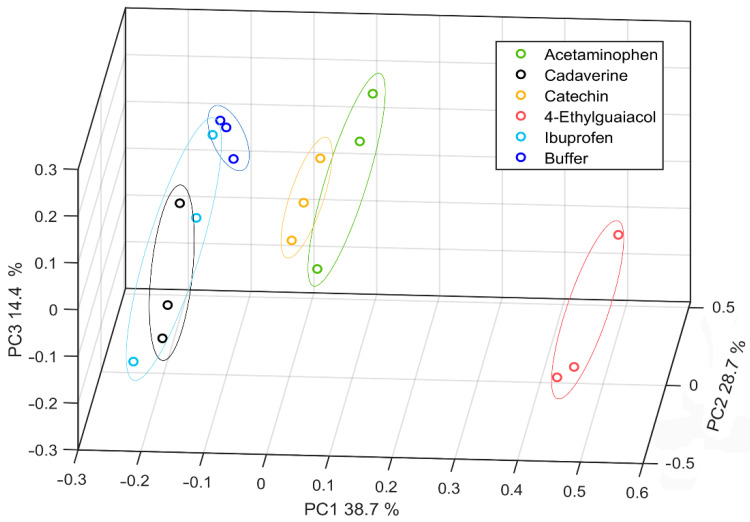
PCA Score plot for the three principal components of 6 different molecules, acetaminophen, cadaverine, catechin, 4-Ethylguaiacol, and ibuprofen.

To visualize the selectivity of the biosensor, a Principal Component Analysis (PCA) (executed in Matlab R2020a) was carried out to show the ability of the biosensor to distinguish between the different substances considered. In particular, six substances from different families were analyzed with a Lac-RGO-modified electrode. As can be seen in Figure 9, the score plot shows that substances without phenols in their molecular structure as ibuprofen and cadaverine have similar behaviors to the buffer response. This fact demonstrates then the correct performance against the specific substrates.

Table 3 was built to compare the results in the presented work with the others in the literature. From here, it can be concluded that our biosensor has a similar behavior to devices with similar properties, even if it is compared with electrodes that include more than one catalytic material.

#### 3.3.3. Kinetic and Inhibition Study

The kinetics in enzymatic studies is one of the most interesting features as it is unique for each enzyme and substrate. For this reason, this issue is normally studied in detail; Leonor Michaelis and Maud Menten developed one of the most common models. This relates the enzyme substrate concentration ([S]) with the reaction rate (V). In this model, the enzyme rate depicts a saturation curve arriving to a steady state situation. At this point, the reaction rate achieves its maximum (V_max_). This typical behavior is clearly observable in Figure 7A and Figure 8B. From here, Michaelis and Menten deduced their equation (Equation (2)), where K_M_ is the Michaelis–Menten’s constant, defined as the concentration of substrate needed to reach half of the maximum rate [71]. It is important to take into account that the K_M_ and V_max_ values will be unique for each pair of enzyme and substrate.

To determine experimentally these parameters, the Lineweaver-Burk linearization is normally used (see its tailored version in Equation (3)) [72]. However, to assure that the Michaelis–Menten kinetics are valid for the studied system, first, the Hills coefficient (h, see Equation (4)) is commonly calculated. In this way, when h results are being close to 1, a Michaelis–Menten model can be assumed.

In this work, the h estimated value from the catechol calibration curve was 0.99 ± 0.09, demonstrating that the Lac-RGO biosensor followed the already explained kinetics. The rate parameters K_M_ and V_max_, obtained from Equation (4), were 2.62 ± 0.50 µmol·L^−1^ and 1.90 × 10^−4^ ± 0.13 × 10^−4^ A, respectively.

(2) $$V_{0}=\;V_{\max}\;\frac{\left[S\right]}{\left[S\right]+K_{M}}$$

(3) $$\frac{1}{V}=\frac{K_{M}}{V_{\max}}\cdot \frac{1}{\left[S\right]}+\frac{1}{V_{\max}}$$

(4) $$V=\frac{V_{\max}\cdot {\left(\left[S\right]/K_{M}\right)}^{h}}{1+{\left(\left[S\right]/K_{M}\right)}^{h}}$$

As commented in the introduction section, there are common household products that have been used for a long period of time, that are now under scrutiny due to their possible effects on the environment and health. Examples are the emerging pollutants found in cosmetics, processed food, or pharmaceutical compounds. The inhibition study of the Lac-RGO biosensor was carried out using benzoic acid and EDTA, two examples of CECs. Benzoic acid is a colorless crystalline compound that is widely used in pharmaceutical products either as an active product ingredient (for its antifungal properties) or as an excipient (as a lubricant in tablets). Both in the food and cosmetics industry, it is used as a preservative [73,74]. On the other hand, EDTA is a colorless and soluble product used in various and distinct applications; for example, in the paper industry as a metal chelate agent or in the food and cleaning industries as a preservative [75,76]. Regarding the legislated limits for the herein target compounds, there is not an individual value. Nevertheless, the WHO made preliminary studies that recommend as maximum values 5 mg·kg^−1^ for the benzoic acid and 600 µg·L^−1^ for the EDTA (values for drinking-water) [77,78].

Any substance that in a particular context can decrease the kinetics of an enzyme will be considered as an enzymatic inhibitor. As mentioned before, the Michaelis–Menten constants (Equation (2)) are unique for a specific enzyme-substrate system, so the presence of inhibitors will lead to renaming the K_M_ and V_max_ to K’_M_ and V’_max_.

There are three modelled scenarios [23]. The first situation is competitive inhibition, where the inhibitor and the substrate compete for the same active site; thus, K_M_ < K’_M_ and V_max_ = V’_max_. The second situation is named non-competitive inhibition; in this case, the inhibitor is not a direct competitor but is bound in an allosteric site that deactivates the enzyme. The changes in the constants are noted as K_M_ = K’_M_ and V_max_ > V’_max_. Finally, the last scenario is the uncompetitive inhibition, where the inhibitor is bound, producing a bigger affinity between the substrate and the enzyme, leading to fewer active sites in the system, in that case K_M_ > K’_M_ and V_max_ = V’_max_ [72].

**Figure 10:**
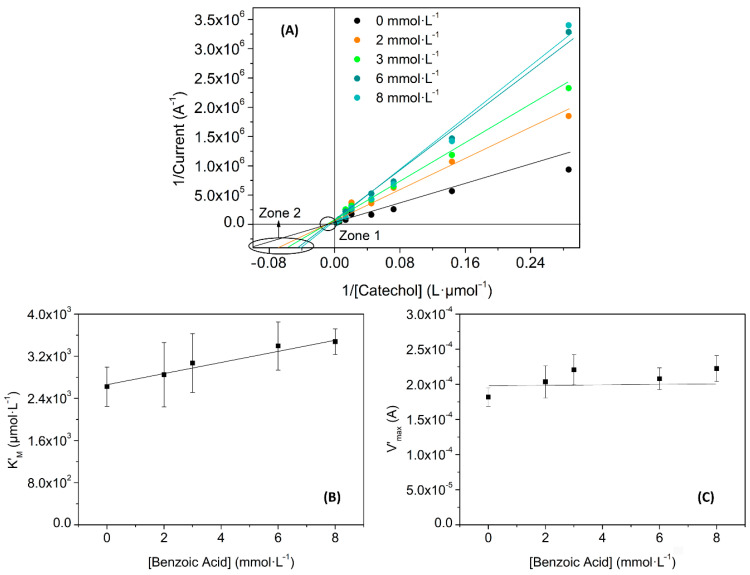
Biosensors kinetic study, (**A**) Lineweaver–Burk linearization for four benzoic acid concentrations; (**B**) representation of the obtained K’_M_ values for each inhibitor concentration; and (**C**) V’_max_ obtained values for each inhibitor concentration.

**Figure 11:**
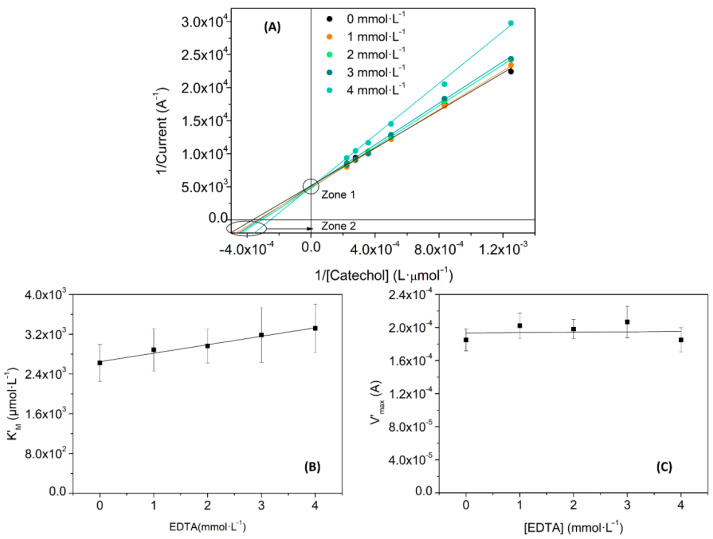
Biosensors kinetic study, (**A**) Lineweaver–Burk linearization for four EDTA concentrations; (**B**) representation of the obtained K’_M_ values for each inhibitor concentration; and (**C**) V’_max_ obtained values for each inhibitor concentration.

Experimentally, the changes can be elucidated comparing the Lineweaver-Burk linearization for different concentrations of inhibitor. In this sense, Figure 10 and Figure 11 show the inhibition effects of different concentrations of benzoic acid and EDTA in a Lac-RGO electrode. Both figures show a typical behavior of the first scenario: a competitive inhibition. Figure 10A and Figure 11A depict the calibration curves of catechol using a Lac-RGO electrode in the presence of different concentrations of benzoic acid and EDTA, respectively. Zone 1 highlighted the steady V_max_ and zone 2 in the increase in K_M_ with the inhibitor concentration. In Table 4 there are complementary data used to corroborate the conclusions from Figure 10A and Figure 11A; concretely, it includes the slopes from the K’_M_ and V’_max_ (calculated from the linear regressions on Figure 10A and Figure 11A), when inhibitor concentrations are varied. In this table, positive slopes confirm the Michaelis constant increase (significant variation of K’_M_ with slope values above uncertainties), and the constant trend for the maximum velocity (the uncertainties include the zero-slope value). This state is supported by Figure 10B,C and Figure 11B,C, where the calculated K_M_’ and V_max_’ values, and their trends, are plotted. 

Moreover, the inhibition constant (K_I_) for each inhibitor. Table 4 shows the higher affinity of benzoic acid (25 mmol·L^−1^) to Laccase than EDTA (17 mmol·L^−1^). To calculate K_I_ values, expression (5), corresponding to competitive inhibition, was used.

(5) $${K^{\prime }}_{M}=K_{M}\cdot \left(1+\frac{\left[I\right]}{K_{I}}\right)$$

**Figure 12:**
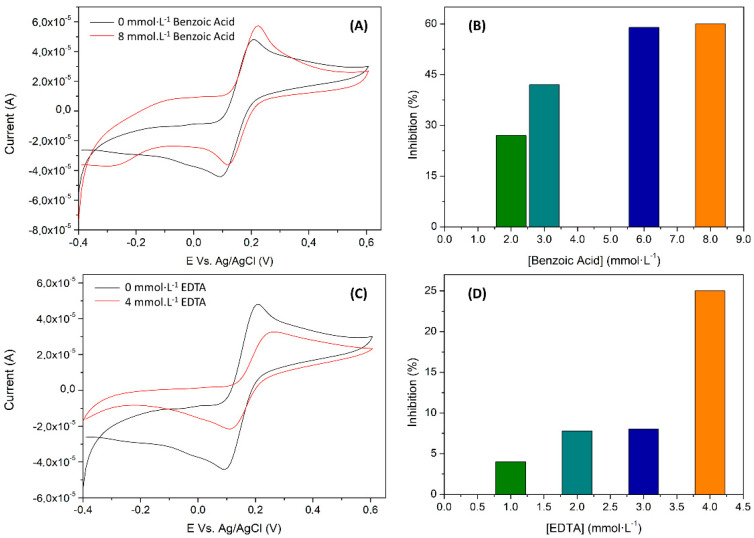
Biosensor inhibition study in a constant concentration of catechol (800 µmol·L^−1^) when different concentrations of (**A**,**B**) benzoic acid and (**C**,**D**) EDTA inhibitors are present in the sample. (**A**,**C**) depicts the CV obtained for the maximum inhibition percentage and (**B**,**D**) the inhibition percentage profile when varying the inhibitor concentration. Percentage of inhibition calculated according to the relative maximum rate velocities, 100·(1 − V’/V).

On a further visualization of the inhibition effect, Figure 12B,D shows the percentage of the inhibition achieved for each pollutant in 800 µmol·L^−1^ of catechol. A bigger effect for benzoic acid, as expected from the previous comments, was fulfilled. In detail, a maximum ≈ 60% of inhibition was reached for the benzoic acid system in front of the ≈25% for EDTA. Voltammograms presented in Figure 12A,C shows the biosensor response in front of the catechol in the presence or absence of the inhibitor. In each case, the shape of the RGO voltammogram profile is recovered (Figure 7B) when benzoic acid and EDTA are in solution, indicating that laccase has lost its activity when they are present. To sum up, the expected inhibition effect was verified, permitting the quantification of EDTA and benzoic acid in controlled samples.

## 4. Conclusions

The reported work has shown the capabilities of graphene as a nanomaterial to improve and facilitate the development of electrochemical biosensors, specifically those employing redox enzymes. The advantages observed can be attributed to two factors: the first one is the amelioration of the electrochemical transduction properties, thanks to inherent electrical characteristics of graphene. The second, the conjunction with the chemical properties of the material, as it serves as a linkage platform for immobilization of biomolecules such as enzymes.

All this makes this combination an unbeatable choice for electrochemical biosensing. The case specified in here, the biosensing of catechol, has demonstrated an improvement of sensitivity of ca. 30%, which in turn improves the direct determination of phenolic compounds or opens the door to consider inhibition. In this inhibitory biosensing application, two different chemicals, EDTA and benzoate, have been verified as inhibitors of the enzyme reaction, which may constitute the basis for a future analytical device or system to monitor CECs in water.

## Figures and Tables

**Table 1:** XPS-obtained distributions for the synthetized GO for the C 1s spectra.

Bond	Energy (eV)	% Area (RSD) *
C=C	284.5	42.81 (1.30%)
C-C	286.4	40.39 (2.1%)
C-O	286.9	10.24 (8.3%)
C=O	288.2	6.56 (9.5%)

* Relative standard deviation.

**Table 2:** Fitted values of circuit elements in the equivalent electric circuit for the different studied surfaces.

Electrode	Rs (Ω)	Rct (Ω)	CPE (F)
GEC	240.7 ± 4	1638 ± 62	7.9 × 10^−5^ ± 0.7 × 10^−5^
RGO	252.0 ± 3	206 ± 142	4.2 × 10^−5^ ± 0.5 × 10^−5^
Laccase-RGO	252.0 ± 2	231 ± 138	4.3 × 10^−5^ ± 0.4 × 10^−5^

**Table 3:** Reported values for the analytical study of sensors and biosensors for the quantification of catechol.

Method	Electrode	LOD (µM)	Sensitivity (µA·µM^−1^)	Linear Range (µM)	Ref
CV	Lac/rGO/GEC	2.1	0.431 (15.39 µA µM^−1^ cm^−2^)	2–100	-
CV	Tyrosinase-polysaccharide	6	0.001	60–800	[60]
CV	Lac/PANI/GCE ^1^	2.07	0.23	3.2–19.6	[55]
CV	Lac/AP-rGOs/GCE ^2^	7	15.79	15–700	[61]
CV	Lac/AA/LuPc2 ^3^	0.58	0.36	4–150	[62]
CV	AuNPs/ZnO/Al2O3/GO/chitosan/GCE	3.1	-	0.5–40	[63]
CV	FePP/MWCNT/Nafion/GCE ^4^	3.75	0.1867	65–1600	[64]
Amp. ^11^	N-OMC/PVA/Lac/AuE ^5^	0.31	0.29	0.39–8.98	[65]
Amp.	Lac/CNTs–CS/GCE ^6^	0.66	-	1.2–30	[66]
Amp	Lac/PAP/MWCNTs/SPE ^7^	0.20	5.8 × 10^4^	0.63–20.7	[47]
Amp	Lac/Nafion/MWCNTs/SPE	0.45	1.6 × 10^4^	1.4–65.4	[47]
Amp	Lac/MWCNTs/SPE	0.73	1.5 × 10^4^	2.4–134.4	[47]
DPV	Lac/MWCNT-COOH/AuNPs/SDBS/PEDOT/GCE ^8^	12.26	0.012	12.0–94.1	[67]
DPV	RGO-MWCNT/GCE	1.8	0.07	5.5–540	[68]
DPV	MWCNT-PMG/GCE ^9^	5.8	1.3 µA µM^−1^ cm^−2^	30–1190	[69]
DPV	MWCNT/PMT/GCE ^10^	0.05	1.895	0.5–150	[70]

^1^ Laccase/Polyaniline/Glassy carbon electrode. ^2^ Lac/aminopyrene-reduced graphene oxide/GCE ^3^ Lac/arachidic acid/Lutetium bisphthalocyanine ^4^ Iron porphyrins/multi-walled carbon nanotubes/Nafion/GCE ^5^ Nitrogen-doped ordered mesoporous carbon/Polyvinyl alcohol/Lac/Au electrode ^6^ Lac/Carbon nanotubes-chitosan/GCE ^7^ Lac/Polyazetidine prepolymer/MWCNTs/Screen printed electrode ^8^ Lac/MWCNT-COOH/AuNPs/sodium dodecyl benzene sulfonate/Poly(3,4-ethylenedioxythiophene)/GCE ^9^ MWCNT- poly(malachite green)/GCE ^10^ MWCNT/ poly (3-methylthiophene)/GCE ^11^ Amperometry.

**Table 4:** Variation of kinetic parameters vs. inhibitor concentration. Values of the linear trend K’_M_ and V’_max_ vs. Inhibitor concentration when kinetics of the laccase enzyme is evaluated. Inhibition values from calibration curves of catechol in different concentrations of acid benzoic and EDTA inhibitors.

Substance	Slope of K’_M_ (µmol·L^−1^) vs. [I]	Slope of V’_max_ (A·L·mmol^−1^) vs. [I]	K_I_ (mmol·L^−1^)
Benzoic acid	0.11 ± 0.04	3·10^−6^ ± 6·10^−6^	25 ± 19
EDTA	0.2 ± 0.1	0 ± 1·10^−5^	17 ± 9

## Data Availability

Not applicable.
